# Plant Growth-Promoting Rhizobacteria Improve Growth, Morph-Physiological Responses, Water Productivity, and Yield of Rice Plants Under Full and Deficit Drip Irrigation

**DOI:** 10.1186/s12284-022-00564-6

**Published:** 2022-03-14

**Authors:** Taia A. Abd El-Mageed, Shimaa A. Abd El-Mageed, Mohamed T. El-Saadony, Sayed Abdelaziz, Nasr M. Abdou

**Affiliations:** 1 Soil and Water Department, Faculty of Agriculture, Fayoum University, Fayoum, 63514 Egypt; 2 Agronomy Department, Faculty of Agriculture, Fayoum University, Fayoum, 63514 Egypt; 3 Department of Agricultural Microbiology, Faculty of Agriculture, Zagazig University, Zagazig, 44511 Egypt; 4 Department of Agricultural Microbiology, Faculty of Agriculture, Fayoum University, Fayoum, 63514 Egypt

**Keywords:** PGPR, Chlorophyll fluorescence, Air–canopy temperature (Tc–Ta), Rice, Water relations, Antioxidant system, Yields

## Abstract

Inoculating rice plants by plant growth promoting rhizobacteria (PGPR) may be used as a practical and eco-friendly approach to sustain the growth and yield of drought stressed rice plants. The effect of rice inoculation using plant growth hormones was investigated under drip full irrigation (FI; 100% of evapotranspiration (ETc), and deficit irrigation (DI; 80% of ETc) on growth, physiological responses, yields and water productivities under saline soil (ECe = 6.87 dS m^−1^) for 2017 and 2018 seasons. Growth (i.e. shoot length and shoot dry weight), leaf photosynthetic pigments (chlorophyll ‘a’ and chlorophyll ‘b’ content), air–canopy temperature (Tc–Ta), membrane stability index (MSI%), and relative water content, (RWC%) chlorophyll fluorescence (F_v_/F_m_) stomatal conductance (gs), total phenols, peroxidase (PO), polyphenol oxidase (PPO), nitrogen contents and water productivities (grain water productivity; G-WP and straw water productivity; S-WP) were positively affected and significantly (*p* < 0.05) differed in two seasons in response to the applied PGPR treatments. The highest yields (3.35 and 6.7 t ha^−1^ for grain and straw yields) as the average for both years were recorded under full irrigation and plants inoculated by PGPR. The results indicated that under water scarcity, application of (I_80_ + PGPR) treatment was found to be favorable to save 20% of the applied irrigation water, to produce not only the same yields, approximately, but also to save more water as compared to I_100%_.

## Introduction

Rice is a very important cereal crop worldwide, supplying more than 50% of the global food demand. The Global rice production was more than 700 × 10^6^ tons year^−1^, produced from 167million ha (FAOSTAT 2018). More than 75% of rice production is supplied by irrigated lowland rice (Ram et al. 2003; Yuan et al. 2021). Generally, rice has been grown under flooded conditions with maintaining a continuous water depth of 5–10 cm (Bouman et al. 2007). Lowland rice mainly is direct-seeded or transplanted in puddled soils by plowing under saturated water conditions, and then followed by harrowing and leveling management. Under flooded conditions, a large amount of irrigation water supply is required, which is not only used to cope with water needs for the growth and development of rice plants but also as a management technique during rice cultivation (Brown et al. 1977; McCauley 1990; Sivapalan, 2015). The irrigation water demand for rice plants under the traditional flooded system is more than 20,000 m^3^ ha^−1^ which is more than 3–4 times that of its biological needs from water (Tuong et al. 2005; Kruzhilin et al. 2015). In a puddled rice field the consumption of water depends on the rates of evaporation, transpiration, and water losses by percolation, seepage, and surface runoff. Therefore the lower water productivity under irrigated rice conditions is referring to water losses (Abd El-Mageed et al. 2020; Abdou et al. 2021). Soil salinity is abiotic stress that limits both vegetative and reproductive development of grown crops (Abd El-Mageed et al. 2019)**.** Worldwide, more than 800 million hectares of arable land are salt affected (Wang et al. 2011). Plants are induced by salinity that causes ion toxicity, osmotic stress, ion imbalance, mineral deficiencies, physiological and biochemical disruption, consequently, reducing the quality and total yield of the affected crop (Rady et al. 2016).

The availability of irrigation water for agriculture, especially for rice production in many regions of the world, is threatened, not only by the global shortage of water resources (Cai et al. 2020) but also by increasing urban and industrial demand (Boretti and Rosa 2019). Worldwide, the production of rice consumes much water more than that of other crops, it is determined that irrigated rice consumes about 40% of the global water used particularly for irrigation purposes (Bouman et al. 2007; Hoekstra et al. 2011).

In Egypt, after wheat, rice ranked a second staple food and cultivated in reclaimed saline soils, in North delta and coastal areas, rice consumes about 10 billion m^3^ of water which is about 18% of the Egyptian share of water from the Nile River. Egypt like many countries of the world faces several challenges respecting the increasing water demand and increasing water competition among users, the sustainability of rice production in Egypt is becoming more threatened by the limited water resources (Abd El-Mageed et al. 2020; Abdou et al. 2021). Therefore, the Ministry of Irrigation and Water Resources in Egypt annually reduces the allotted area for rice cultivation, which is decreased by 59% from 745,000 ha to 304,080 ha during the past 10 years (2008–2018).

Water stress negatively affects the growth and productivity of crops (Ahuja et al. 2010; Shekoofa and Sinclair 2018). Physiological functioning in rice plants (Guimarães et al. 2013; Yang et al. 2019; Abdou et al. 2021) viz root length density, root moisture extraction, the rate of apical development, canopy size, leaf elongation rate, leaf rolling, transpiration rate, RWC, biomass production, spikelet number, spikelet sterility, panicle development, grain size, and grain yield (Palanog et al. 2014; Kruzhilin et al. 2016; Yang et al. 2019) may be drastically reduced due to water stress, especially if it occurs during vegetative or reproductive stages of rice, depending upon the stress severity and cultivar tolerance. In recent years, the trickle irrigation system has been spread out more intensely, not only for enhancing water productivity but also for increasing crop production (Geerts and Raes 2009). Drip irrigation can achieve application efficiencies as high as 90% if the system is well maintained and combined with soil moisture monitoring or other ways of assessing crop water requirements (Vickers 2002; Jägermeyr et al. 2015). Water use efficiency and crop production can be enhanced by using drip irrigation under limited water resources by declining the volume of water that leaches out of the root zone (El-Hendawy et al. 2008). Irrigation techniques that tend to minimize the inputs of irrigation water for rice production like deficit irrigation should be applied. Deficit irrigation (DI) is a method mainly applied to decrease water losses and maximize water productivity (WP), particularly in areas where the water supply is inadequate for irrigation (Agami et al. 2018; Abd El-Mageed et al. 2019; Semida et al. 2020). DI can also have other benefits related to reducing the energy used during irrigations and decreasing nitrate leaching (Falagán et al. 2015), reducing production costs and water consumption (Badal et al. 2013; Ballester et al. 2014).

To cope with drought stress, several adaptations and strategies are required. Plant growth-promoting rhizobacteria (PGPR) could play a significant role in the alleviation of induced injurious effects by drought stress on plants (Vurukonda et al. 2016). The role of microorganisms regarding plant growth, nutrient management, and biocontrol activity is very well established. These beneficial microorganisms colonize the rhizosphere/endo-rhizosphere of plants and promote the growth of the plants through various direct and indirect mechanisms (Grover and Ali 2011). Furthermore, the role of microorganisms in the management of biotic and abiotic stresses is gaining importance. The possible explanation for the mechanism of plant drought tolerance induced by rhizobacteria includes (1) production of phytohormones like abscisic acid (ABA), gibberellic acid, cytokinins, and indole-3-acetic acid (IAA); (2) ACC deaminase to reduce the level of ethylene in the roots; (3) induced systemic tolerance by bacterial compounds; (4) bacterial exopolysaccharides (Timmusk et al. 2014; Carlson et al. 2020; Getahun et al. 2020; Poudel et al. 2021). Hence, the application of PGPR may increase water-saving and enhance crop yield productivity under conditions of deficit water supply. Likewise, rice crop responses to combined PGPR with deficit irrigation regimes synchronized with salt affected soils have not yet been investigated. Therefore, the main objective of the current study was to investigate the effect of PGPR application and DI on growth, plant defense system, physio-biochemical attributes, seed and straw yield, and WP of rice plants cultivated in salt soil-affected.

## Materials and Methods

### Experimental Set-Up

Our study was conducted in the private farm South-east Fayoum, (29° 35′ N; 30° 05′ E) Egypt for two successive years 2017 and 2018. The climate is arid, characterized by low precipitation and rainfall occurs mainly during the period from December to April. The region is also characterized by more than 320 days a year of sunny days. The meteorological parameters (i.e. air temperature °C, relative humidity (%), wind speed (m s^−1^) and pan evaporation (mm day^−1^) during the rice cultivation period in 2017 and 2018 were presented in (Table 1). The soil, 80–100 cm deep, is loamy sand and defined as Typic Torripsamments, siliceous, hyperthermic (Soil Survey Staff 1999). Physio-chemical characteristics of the soil were: pH 7.85 (1:2.5 soil/water extract), Kjeldahl total N 1.4 g kg^−1^, Olsen extractable P 3.53 mg kg^−1^, ammonium acetate extractable K 42.85 mg kg^−1^, organic C 8.2 g kg^−1^, total carbonate 43.7 g kg^−1^, ECe (soil paste extract) 6.4 dS m^−1^, bulk density 1.53 kg dm^−3^, field capacity and wilting point 21.31% and 10.3%, respectively Tables 2 and 3.

**Table 1:** The climatic data recorded at Meteorological observatory of Fayoum governorate, during crop growing seasons of 2017 and 2018

Month	Year	Temperature °C			Relative humidity (%)	Wind speed (m s^−1^)	Pan evaporation (mm day^−1^)
		Max	Min	Mean			
June	2017	36.0	21.7	28.9	42	5.2	7.3
	2018	40.3	24.4	32.3	38	5.0	7.5
July	2017	37.0	21.8	29.4	35	4.0	6.9
	2018	39.3	23.9	31.6	37	3.7	6.9
August	2017	40.4	26.0	33.2	36	1.9	6.2
	2018	36.4	23.0	29.6	45	3.7	6.3
September	2017	38.3	13.8	31.0	36	2.0	5.5
	2018	35.3	21.0	28.0	44	3.5	5.3

**Table 2:** Some initial physical properties of the experimental soil

Layer (cm)	Particle size distribution				Bulk density (g cm^−3^)	K_sat_ Cm h^−1^	FC (%)	WP (%)	AW (%)
	Sand%	Silt%	Clay%	Texture class					
0–25	79.2	10. 0	10.8	LS	1.58	2.22	24.33	10.73	14.60
25–50	77.2	10.1	10.7	LS	1.55	1.55	22.19	12.13	10.06

FC = Field capacity, W.P = wilting point, A.W = Available water, LS = loamy sand and K_sat_ = Hydraulic conductivity

**Table 3:** Some initial chemical properties of the experimental soil

Properties	Value
pH [at a soil: water(w/v) ratio of 1:2.5]	7.86
ECe (dS m^−1^; soil—paste extract)	6.12
CEC(cmol_e_ kg^−1^)	11.10
CaCO_3_ (%) Organic matter (%) ESP (exchangeable sodium percentage)	4.81
	1.10
	10.62
SAR	12.07
*Available nutrients*	
N (%)	0.05
P (mg kg^−1^ soil)	5.28
K (mg kg^−1^ soil)	69.9
Fe (mg kg^−1^ soil)	3.4
Mn (mg kg^−1^ soil)	10.6
Zn (mg kg^−1^ soil)	0.7
Cu (mg kg^−1^ soil)	0.5

### Experimental Design and Plant Management

Two field experiments were conducted in a randomized complete block design (Split Plot). 2 irrigation treatments were applied (100, and 80% of ETc were occupied as main plots) and two PGPR treatments (treated and non-treated were allocated to sub-plots). The 4 treatments were replicated three times, making a total of 12 plots. The area of the experimental plot was 16 m length × 0.8 m row width (12.80 m^2^), each plot included 4 planting rows placed 20 cm apart with a distance of 10 cm between plants within rows. Two drip lines were placed 0.40 m apart in each elementary test plot. Healthy seeds of rice (*Oryza sativa* L.), variety Sakha 107 were sown on 20 May 2017 and 2018. The 4-week-old transplants were transported and replanted and then harvested on 6 October 2017 and 2018. Mineral fertilization, pest management, disease, and cultural practices were performed as the instructions of local commercial crop production. Irrigation water applied (IWA) was estimated as a percentage of the crop evapotranspiration (ETc) representing the following three treatments: FI = 100%, and DI = 80% of ETc. Daily ETo and ETc were estimated according to Allen et al. (1998) equation.

$${\text{IWA}} = \frac{{{\text{A }} \times {\text{ETc}}}}{{{\text{Ea}} \times 1000{ } \times \left( {1 - {\text{LR}}} \right)}}$$

where IWA: irrigation water applied (m^3^), A: irrigated plot area (m^2^), ETc: water consumptive use (mm day^−1^) and was computed as follow;

$${\text{ETc}} = {\text{ETo}} \times {\text{Kc}}$$

ETo is the reference evapotranspiration (mm d^−1^) and Kc = crop coefficient. ETo was determined as follows:

$${\text{ETo}} = {\text{Epan}} \times {\text{Kp}}$$

where Epan is the evaporation from a class A and Kp is the pan coefficient, Ea: efficiency of application (%) and LR: leaching requirements.

### Growth and Physiological Measurements

At the tillering stage of both seasons/experiments, 5 individual plants were randomly chosen from each experimental plot to evaluate growth characteristics and another group of 5 plants to determine chemical attributes. Shoot lengths and spikes lengths were measured using a meter scale. The number of spikes was counted per plant, and leaf area per plant was measured using Digital Planimeter (Planix 7). Shoots of plants were weighed to record their fresh weights and then placed in an oven at 70 ± 2 °C till a constant weight to measure their dry weights.

### Chlorophyll Fluorescence (F_v_/F_m_) and Performance Index (PI)

The (F_v_/F_m_) was measured by using a portable fluorometer (Handy PEA, Hansatech Instruments Ltd, Kings Lynn, UK) and calculated according to Maxwell and Johnson (2000). Where the (PI) of photosynthesis based on equal absorption (PI_ABS_) was calculated as reported by Clark et al. (2000).

### Stomatal Conductance (gs) and Leaf Chlorophyll Concentration (SPAD)

The gs was measured on fully expanded upper canopy leaves between 10 and 12 h with a portable photosynthetic system (CIRAS-2, PP Systems, Hitchin, UK). The SPAD was determined at 90 DAS for the three youngest completely expanded leaves per hill by (SPAD-value; SPAD502, KONICAMINOLTA. Inc., Tokyo).

### Rice Water Status (RWC %, MSI %, and Canopy Temperature)

The RWC was determined according to Hayat et al. (2007) equation as follows;

$${\text{RWC}}\,(\% ) = \left[ {\frac{{\left( {{\text{FM}} - {\text{DM}}} \right)}}{{\left( {{\text{TM}} - {\text{DM}}} \right)}}} \right] \times 100$$

where RWC% is relative water content (%), FM: fresh mass (g), TM: turgid mass (g), and DM is the dry mass (g). Likewise, MSI% was determined and calculated using the method of Premachandra et al. (1990) as follow

$${\text{MSI}}\left( {\text{\% }} \right) = \left[ {1 - \frac{C1}{{C2}}} \right] \times 100$$

where MSI % is the membrane stability index, C_1_: is the EC of the solution at 40 °C and C_2_: is the EC of the solution at100°C. Canopy temperature (Tc) was measured by a hand-held infrared thermometer (Fluk 574, Everett WA, USA) at an emissivity of 0.98 and a spectral response range of 8–14 µm.

### Total Nitrogen and Antioxidant Defense System

Total nitrogen was determined according to the well-known method described by Donald and Robert (1998). Estimations of total phenols, peroxidase (PO), and polyphenol oxidase (PPO) were carried out by the method described by Ramamoorthy et al. (2002).

### Chlorophyll ‘a’ and Chlorophyll ‘b’ Content

Chlorophyll ‘a’ and chlorophyll ‘b’ content were extracted and determined (in mg g^−1^ FW) according to the procedure given by Arnon (1949) using a UV-160 A UV–Vis recording spectrometer (Shimadzu, Kyoto, Japan) at 663 and 645 nm.

### Rhizobacteria Strains Preparations and Inoculation of Rice Seedlings

The most effective facultative oligotrophic bacterial two strains used in this experiment as PGPR were isolated from the same soils at Fayoum region, Egypt, and were completely identified as [*Bacillus subtilis subsp, spizizenii strain NRRL B-23049T and Bacillus megatherium strain IAM 13418].* The most effective facultative oligotrophic bacterial strains obtained, from the previous part, were selected and chosen for some different characters based on previous knowledge of their ability to produce (indole acetic acid IAA, Salicylic acid, zinc, and phosphate solubilization, N_2_-fixation, cellulase and chitinase, oxidase, catalase activities and lactose fermentation (Table 4).

**Table 4:** Morphological, physiological and biochemical characters

Characteristic/strain	*Bacillus megaterium* CCMMB583	*Bacillus subtilis* KJB06
Gram stain	+	+
Motility	+	+
Spore forming	+	+
Growth at: 10, 20, 30, 40 and 50 °C	+	+
Indole production	+	+
Citrate utilization	+	+
*Fermentation of*		
D-glucose	+	+
Lactose	-	+
D-mannitol	+	-
D-xylose	+	+
*Growth in NaCl*		
5%	+	+
10%	+	+
15%	+	+
pH 6, 8, 10 and 12	+	+
*Metabolites and hydrolytic enzymes*		
Oxidase	+	+
Catalase	+	+
Gelatin hydrolysis	+	+
Starch hydrolysis	+	+
Cellulose hydrolysis	+	+
Chitinase	+	+
*Activities related to plant growth promotion*		
Siderophore production	+	+
Indoleacetic acid production	+	+
Salicylic acid production	+	+
Phosphate solubilization	+	+
Zinc solubilization	+	+
Putative nitrogen fixation	+	+
*Antifungal activity (inhibition zone mm)*		
Pythium ultimum	16.5	20
Rhizoctonia solani	15.0	18
Fusarium oxysporum	18	21
Phytophthora infestans	21	17

For the preparation of bacterial strains inoculants (antagonizers), each strain was grown individually on sterilized nutrient broth medium in flasks with 1 L capacity on rotary shaker after shaking for 72 h incubation period at 30 °C. The growing organisms were concentrated by centrifuging the medium and cell sidements were aseptically collected and diluted, by the same medium, to 250 mL only (1/4 L). In the case of using a mixture of the two antagonizers, an equal volume of the three strains was mixed instantaneously before use. 20 mL of the resultant suspension was poured twice directly onto the rice seedlings in cones at the seedling and at 15 days after transplanting.

### Water Productivities

*Water productivities* as mentioned byFernández et al. (2020) were calculated as (1) the ratio between above-ground biomass and crop evapotranspiration, i.e. straw WP (S-WP) and (2) the ratio between grain yield and crop evapotranspiration, i.e. grain WP (G-WP) according to Jensen (1983).

### Statistical Analysis

Statistical analysis was performed through the procedure of GenStat (version 11) (VSN International Ltd, Oxford, UK). The least significant difference (LSD) at 5% probability (*p* ≤ 0.05) level was used as mean separation test.

## Results

### Rice Growth in Response to Plant Growth Promoting Bacteria Under Full and Deficit Irrigation

Data in Table 5 illustrate the effects of irrigation level, plant growth promoting bacteria, and their interaction on rice growth. Plants under deficit irrigation had lower growth traits (i.e. shoot length, tillers number plant^−1^, panicles number plant^−1^ and shoot dry weight) than those under full irrigation. On the other hand, plants treated with PGPR had higher growth traits (i.e. shoot length, tillers number plant^−1^, panicles number plant^−1^ and shoot dry weight) than untreated plants. Growth traits were decreased significantly with increasing water stress, I_80%_ resulted in decreases of plant height by 8%, tillers number by 11.8%, panicles number by 12.4%, and shoot dry weight by 25% as compared to fully irrigated plants. On the other hand, treated rice plants with PGPR increased significantly these parameters by 9.4%, 15.3%, 18.6%, and 29.6% for plant height, tillers number, panicles number, and shoot dry weight, respectively. The combined application of PGPR and irrigation at 100% of ETc recorded the best growth parameters, while the treatment I_80_ × ^−^PGPR showed the lowest values of growth parameters_._ Otherwise, no significant differences were found between I_100_ × ^−^PGPR and I_80_ × ^+^PGPR treatments.

**Table 5:** Effect of integrative deficit drip irrigation and plant growth promoting rhizobacteria on growth characteristics of rice plants grown under saline soil for (SI) 2017 and (SII) 2018 seasons

Source of variation	Shoot length (cm)	Tillers no. plant^−1^	Panicles no plant^−1^	Shoot dry weight (g)
Season	NS	NS	NS	NS
(SI) 2017	53.44 ± 1.20a	1.92 ± 0.23a	1.79 ± 0.15a	3.45 ± 0.33a
(SII) 2018	53.66 ± 0.99a	1.95 ± 0.23a	1.77 ± 0.21a	3.47 ± 0.43a
Irrigation	**	*	**	**
FI	55.62 ± 1.20a	2.04 ± 0.32a	1.88 ± 0.23a	3.84 ± 0.45a
DI	51.49 ± 0.89b	1.83 ± 0.54b	1.67 ± 0.30b	3.08 ± 0.32b
PGPR	**	*	**	**
^−^PGPR	51.16 ± 0.88b	1.80 ± 0.31b	1.63 ± 0.29b	3.01 ± 0.28b
^+^PGPR	55.94 ± 0.96a	2.07 ± 0.33a	1.93 ± 0.31a	3.91 ± 0.43a
I × PGPR	**	*	**	**
I_100_ × ^−^PGPR	53.04 ± 1.40b	1.88 ± 0.41b	1.71 ± 0.25b	3.38 ± 0.31b
I_80_ × ^−^PGPR	49.28 ± 1.21c	1.72 ± 0.21c	1.54 ± 0.43b	2.65 ± 0.41c
I_100_ × ^+^PGPR	58.20 ± 0.42a	2.21 ± 0.33a	2.05 ± 0.21a	4.31 ± 0.59a
I_80_ × ^+^PGPR	53.69 ± 0.98b	1.94 ± 0.23b	1.81 ± 0.40b	3.51 ± 0.36b

*,**respectively, differences at *p* ≤ 0.05 and *p* ≤ 0.01 probability level, ns indicates no significant difference. Means followed by the same letter in each column are not significantly different according to the LSD test (*p* < 0.05)

### Rice Water Status

Results of rice water states (RWC, MSI, and canopy-air temperature) in responses to irrigation and PGPR treatments and their interaction are presented in Table 6. The water status of rice plants as evaluated by RWC, MSI, and the canopy-air temperature was significantly affected by irrigation treatment. Data in (Table 6) reflected that RWC and MSI of well-irrigated plants were higher (82.3 and 75.3) than those under deficit irrigation (70.8 and 66.5). On contrary, canopy-air temperature (Tc–Ta) at 13.0 and 14 O’clock of plants irrigated at 100% of ETc (1.24 and 1.59) was lower than plants irrigated at 80% of ETc (1.97 and 2.08). Also, values of RWC, MSI, and the canopy-air temperature were affected positively or negatively by PGPR inoculation. The values of RWC and MSI% for plants treated with PGPR (82.1 and 75.6) were higher than ^−^PGPR plants (63.5 and 73.8). Interaction between PGPR and irrigation treatment significantly affected plant water status. According to the results in Table 6, No significant effects were observed between seasons on RWC, MSI, and Tc–Ta.

**Table 6:** Effect of integrative deficit drip irrigation and plant growth promoting rhizobacteria on plant water status (MSI% and RWC%), canopy-air temperature (Tc–Ta) of rice plants grown saline soil for (SI) 2016/2017 and (SII) 2017/2018 seasons

Source of variation	RWC%	MSI%	Tc–Ta	
			O’clock	
			13:00	14.00
Season	NS	NS	NS	NS
(SI) 2017	77.75 ± 2.53a	67.79 ± 2.21a	1.51 ± 0.26a	1.83 ± 0.27a
(SII) 2018	79.90 ± 2.21a	69.49 ± 2.60a	1.70 ± 0.23a	1.86 ± 0.21a
Irrigation	**	**	**	**
FI	82.33 ± 2.20a	70.82 ± 1.12a	1.24 ± 0.14b	1.59 ± 0.22b
DI	75.33 ± 2.32b	66.45 ± 0.98b	1.97 ± 0.28a	2.08 ± 0.26a
PGPR	**	**	**	**
^−^PGPR	75.58 ± 0.79b	63.46 ± 0.77b	2.15 ± 0.09a	2.60 ± 0.07a
^+^PGPR	82.08 ± 2.10a	73.82 ± 2.20a	1.06 ± 0.24b	1.07 ± 0.11b
I × PGPR	**	**	**	**
I_100_ × ^−^PGPR	80.25 ± 2.11b	64.94 ± 1.11c	1.58 ± 0.18b	2.28 ± 0.08b
I_80_ × ^−^PGPR	70.90 ± 1.23c	61.98 ± 1.21c	2.72 ± 0.31a	2.92 ± 0.05a
I_100_ × ^+^PGPR	84.40 ± 2.22a	76.71 ± 1.42a	0.90 ± 0.12c	0.93 ± 0.09d
I_80_ × ^+^PGPR	79.75 ± 2.14b	70.93 ± 1.09b	1.22 ± 0.31bc	1.23 ± 0.03c

*,**Respectively, differences at *p* ≤ 0.05 and *p* ≤ 0.01 probability level, ns indicates no significant difference. Means followed by the same letter in each column are not significantly different according to the LSD test (*p* < 0.05)

### Stomatal Conductance (gs)

**Fig. 1:**
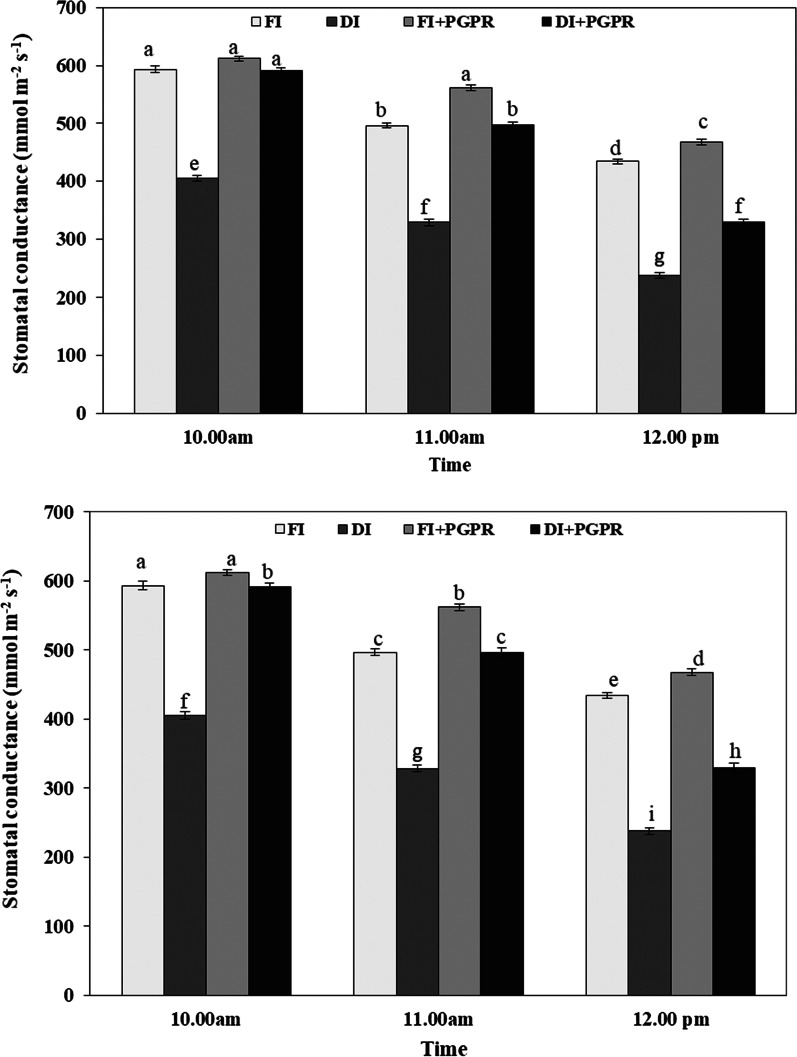
Effect of plant growth promoting bacteria on stomatal conductance of rice plants grown under deficit and non-deficit drip irrigation from 10 am to 12 pm during (SI) 2017 and (SII) 2018 seasons. Error bars indicate standard errors of means (S.E.) (n = 3)

The influences of plant growth promoting bacteria on stomatal conductance (gs) under full and deficit irrigation are presented in Fig. 1. Results showed that gs values were almost stable from 10 to 11 am but thereafter, gs decreased sharply at 12 pm in all treatments. The values of stomatal conductance were higher under FI than those of DI. Maximum values of stomatal conductance were found in FI + PGPR treatment which was greater than those of FI, DI, and DI + PGPR treatments for all times (10 am, 11 am, and 12 pm). Basically, inoculated rice plants increased gs in comparison to the uninoculated control plant.

### Chlorophyll Fluorescence Efficiency, Relative Chlorophyll Content and Photosynthetic Pigments

Responses of chlorophyll fluorescence (Fv/Fm and PI), relative chlorophyll content (SPAD value), and photosynthetic pigments (chlorophyll a and chlorophyll b) of rice plants to irrigation and plant growth promoting bacteria treatments and their interactions are displayed in Table 7. Except for PI, no significant differences were observed between seasons. Chlorophyll fluorescence, relative chlorophyll content, and photosynthetic pigments were significantly influenced by irrigation, PGPR treatments and by their interaction. Results in (Table 7) showed that Fv/Fm, PI, SPAD, chlorophyll "a" and chlorophyll "b" of rice plants under well-watered conditions were compared by water-stressed 7.7, and 14.3%, respectively as compared by water stressed plants. Also, inoculation rice plants by PGPR positively increased Fv/Fm by 5.1%, PI by 66.7%, SPAD by 13.8%, and chlorophyll “a” by 10.5% and chlorophyll “b” by 14.3% as compared with uninoculated plants. Chlorophyll fluorescence, relative chlorophyll content, and photosynthetic pigments were strongly influenced by the interaction between PGPR and irrigation treatments. Maximum values of Fv/Fm, PI, SPAD, chlorophyll a, and chlorophyll b were recorded under I_100_ × ^+^PGPR treatment, while the minimum values for these parameters were observed under I_80_ × ^−^PGPR treatment.

**Table 7:** Effect of integrative of deficit drip irrigation and plant growth promoting rhizobacteria on plant water status (MSI% and RWC %), chlorophyll fluorescence (Fv/Fm and PI) SPAD value, chlorophyll a and chlorophyll a of rice plants grown saline soil for (SI) 2016/2017 and (SII) 2017/2018 seasons

Source of variation	Fv/Fm	Performance index (%)	SPAD chlorophyll	Chlorophyll a	Chlorophyll b
Season	NS	*	NS	NS	NS
(SI) 2017	0.79 ± 0.01a	4.33 ± 0.63a	42.83 ± 1.50a	0.41 ± 0.02a	0.31 ± 0.00a
(SII) 2018	0.80 ± 0.01a	3.75 ± 0.55b	43.55 ± 1.36a	0.39 ± 0.01a	0.29 ± 0.02a
Irrigation	**	**	**	**	**
FI	0.81 ± 0.01a	4.88 ± 0.54a	45.23 ± 0.66a	0.42 ± 0.01a	0.32 ± 0.00a
DI	0.79 ± 0.01b	3.20 ± 0.43b	41.15 ± 1.20b	0.39 ± 0.01b	0.28 ± 0.01b
PGPR	**	**	**	**	**
^−^PGPR	0.78 ± 0.02b	3.03 ± 0.45b	40.40 ± 0.82b	0.38 ± 0.00b	0.28 ± 0.00b
^+^PGPR	0.82 ± 0.01a	5.05 ± 0.32a	45.98 ± 0.76a	0.42 ± 0.01a	0.32 ± 0.02a
I × PGPR	**	**	**	**	**
I_100_ × ^−^PGPR	0.79 ± 0.01b	3.95 ± 0.43b	43.00 ± 0.75b	0.40 ± 0.02b	0.31 ± 0.01ab
I_80_ × ^−^PGPR	0.76 ± 0.02c	2.10 ± 0.24c	37.80 ± 0.53c	0.37 ± 0.00c	0.26 ± 0.00c
I_100_ × ^+^PGPR	0.82 ± 0.01a	5.80 ± 0.24a	47.45 ± 0.51a	0.43 ± 0.01a	0.33 ± 0.02a
I_80_ × ^+^PGPR	0.81 ± 0.01a	4.30 ± 0.32b	44.50 ± 0.67b	0.41 ± 0.01ab	0.30 ± 0.01b

*,**respectively, differences at *p* ≤ 0.05 and *p* ≤ 0.01 probability level, ns indicates no significant difference. Means followed by the same letter in each column are not significantly different according to the LSD test (*p* < 0.05)

### Antioxidant Defense System and Nitrogen Contents

The effects of irrigation, PGPR treatments and their interaction on defense principles like [(peroxidase (PO), polyphenol oxidase (PPO) and total phenol)], N% (leaves) and N% (grains) contents of rice plants were presented in Table 8. The concentration of PO, PPO, total phenol and the content of N% (leaves and grains) were strongly (*p* < 0.05) affected by irrigation quantity and plant growth promoting bacteria and were not positively affected by season except for total phenol. Data in (Table 8) reflected that PO, PPO, total phenol, N content in leaves and grains when rice plants were received 100% of irrigation water requirements were higher by 28.1, 17.7, 7.3, 8.3, and 6.4%, respectively as compared by plants received 80% of ETc. Additionally, rice plants inoculated by PGPR positively increased PO by 20.0%, PPO by 58.3%, total phenol by 24.8%, leaves N content by 33.9%, and grains N content by 20.0% as compared with uninoculated plants. According to the results displayed in Table 8, PO, PPO, total phenol, N content (in leaves, and in grains) were significantly (*p* < 0.05) affected by the interaction between PGPR and irrigation treatments. The highest values of PO, PPO, total phenol, N content in leaves and grains were found when plants were irrigated at 100% of ETc and inoculated by PGPR treatment (I_100_ × ^+^PGPR), while the lowest values for the aforementioned parameters were recoded when rice plants were exposed to water stress (I_80_) and untreated by PGPR (I_80_ × ^−^PGPR).

**Table 8:** Effect of integrative deficit drip irrigation and plant growth promoting rhizobacteria on chlorophyll content (a and b), PO, PPO, total phenol and N% of rice plants grown saline soil for (SI) 2016/2017 and (SII) 2017/2018 seasons

Source of variation	PO	PPO	Total phenol	N% (leaves)	N% (grains)
Season	NS	NS	*	NS	NS
(SI) 2017	0.28 ± 0.01a	0.32 ± 0.00a	1.50 ± 0.20a	2.16 ± 0.23a	1.20 ± 0.04a
(SII) 2018	0.26 ± 0.02a	0.30 ± 0.00a	1.39 ± 0.23b	2.25 ± 0.22a	1.22 ± 0.04a
Irrigation	**	**	**	**	**
FI	0.32 ± 0.01a	0.34 ± 0.01a	1.50 ± 0.22a	2.30 ± 0.17a	1.25 ± 0.04a
DI	0.23 ± 0.01b	0.28 ± 0.00b	1.39 ± 0.11b	2.11 ± 0.21b	1.17 ± 0.03b
PGPR	**	**	**	**	**
^−^PGPR	0.25 ± 0.00b	0.24 ± 0.02b	1.29 ± 0.05b	1.89 ± 0.14b	1.10 ± 0.02b
^+^PGPR	0.30 ± 0.01a	0.38 ± 0.01a	1.61 ± 0.11a	2.53 ± 0.20a	1.32 ± 0.02a
I × PGPR	**	**	**	**	**
I_100_ × ^−^PGPR	0.30 ± 0.01b	0.27 ± 0.01c	1.41 ± 0.23b	1.96 ± 0.31c	1.12 ± 0.01c
I_80_ × ^−^PGPR	0.19 ± 0.02c	0.21 ± 0.00d	1.18 ± 0.13c	1.82 ± 0.23c	1.08 ± 0.03c
I_100_ × ^+^PGPR	0.34 ± 0.01a	0.41 ± 0.02a	1.60 ± 0.14a	2.65 ± 0.28a	1.38 ± 0.01a
I_80_ × ^+^PGPR	0.29 ± 0.01b	0.35 ± 0.01b	1.61 ± 0.21a	2.40 ± 0.36b	1.26 ± 0.02b

*,**respectively, differences at *p* ≤ 0.05 and *p* ≤ 0.01 probability level, ns indicates no significant difference. Means followed by the same letter in each column are not significantly different according to the LSD test (*p* < 0.05)

### Yield Components

Responses of rice yield components such as; panicle length, (cm), panicle weight (g), number of grains panicle^−1^, and 1000 grain weight (g) to cropping seasons, irrigation, PGPR, and their interaction are presented in Table 9. Rice yield components were positively affected by irrigation level, PGPR, and by their interaction and were not affected by the growing season. Yield components of rice plants exposed to drought stress were decreased by 7.5% for panicle length, by panicle weight 23.7%, the number of grains panicle^−1^ 10.8%, and 1000 grain weight of rice plants by 17.8% as compared with unstressed plants. On the other hand, inoculated rice plants by PGPR increased yield component by 10.6, 28.0, 19.9, and 23.0% for panicle length, panicle weight, number of grains panicle^−1^, and 1000 grain weight as compared by untreated plants, respectively. Our results showed that rice yield components were strongly influenced by the interaction between PGPR and irrigation treatments. The highest values of panicle length, panicle weight, number of grains panicle^−1^ and 1000 grain weight (15.8, 2.1, 75.1 and 22.3) were recorded when plants received 100% of ETc and inoculated by PGPR (I_100_ × ^+^PGPR), while the lowest values for aforementioned traits (13.6, 1.2, 56.2 and 14.7) were recorded when rice plants exposed to water stress (I_80_) and untreated by PGPR (I_80_ × ^−^PGPR) treatment.

**Table 9:** Effect of integrative deficit drip irrigation and plant growth promoting rhizobacteria on yield component, grain yield and straw yield of rice plants grown under saline soil for (SI) 2016/2017 and (SII) 2017/2018 seasons

Source of variation	Panicle length (cm)	Panicle weight (g)	≠ grains/panicle	1000 grain weight (g)
Season	NS	NS	NS	NS
(SI) 2017	14.58 ± 1.41a	1.69 ± 0.11a	64.60 ± 2.11a	18.78 ± 1.01a
(SII) 2018	14.72 ± 1.21a	1.73 ± 0.11a	63.39 ± 1.91a	18.80 ± 0.91a
Irrigation	**	**	**	**
FI	14.99 ± 1.12a	1.94 ± 0.11a	67.66 ± 1.45a	20.63 ± 1.89a
DI	13.87 ± 0.88b	1.48 ± 0.09b	60.33 ± 2.31b	16.95 ± 1.21b
PGPR	**	**	**	**
^−^PGPR	13.91 ± 0.74b	1.50 ± 0.13b	58.19 ± 1.63b	16.85 ± 0.66b
^+^PGPR	15.39 ± 1.06a	1.92 ± 0.22a	69.81 ± 1.92a	20.73 ± 1.12a
I × PGPR	**	**	**	**
I_100_ × ^−^PGPR	14.20 ± 1.20b	1.77 ± 0.22b	60.20 ± 2.30c	19.00 ± 1.02b
I_80_ × ^−^PGPR	13.62 ± 0.88c	1.23 ± 0.21c	56.18 ± 1.99c	14.70 ± 0.89c
I_100_ × ^+^PGPR	15.79 ± 1.23a	2.11 ± 0.17a	75.13 ± 2.32a	22.25 ± 1.23a
I_80_ × ^+^PGPR	14.11 ± 0.99b	1.73 ± 0.14b	64.49 ± 2.42b	19.20 ± 1.43b

*,**Respectively, differences at *p* ≤ 0.05 and *p* ≤ 0.01 probability level, ns indicates no significant difference. Means followed by the same letter in each column are not significantly different according to the LSD test (*p* < 0.05)

### Rice Yields and Water Productivities

Table 10 illustrates the effects of growing seasons, irrigation level, PGPR, and their interaction on rice yields (grain and straw; t ha^−1^) and water productivities *(*G-WP, and S-WP; kg m^−3^). Plants grown under full irrigation had higher yields (i.e. grain yield, straw yield) than those grown under drought stress. Grains yield, straw yield, were decreased positively with increasing water stress, I_80%_ resulted in decreases of grain yield by 19%, straw yield by 11.9%, in relation to fully irrigated plants. On the other hand, values of G-WP, and S-WP under I_80%_ treatment were higher than those of I_100%_ treatment by 1.3 and 10.4%, respectively, (Table 10). Rice plants treated with PGPR increased grains yield, straw yield, G-WP, and S-WP by 19.0, 16.8, as compared with untreated plants. No significant differences between growing seasons were observed. Our findings showed that grains yield, straw yield, G-WP, and S-WP were significantly affected by the interaction between PGPR and irrigation treatments. Plants fully irrigated and inoculated by ^+^PGPR gained the highest values of grains yield (5.24 t ha^−1^), straw yield (8.87 t ha^−1^), G-WP (kg m^−3^), and S-WP (kg m^−3^). Moreover, the lowest values for grains yield (3.65 t ha^−1^), straw yield (6.58 t ha^−1^), G-WP (kg m^−3^), and S-WP (kg m^−3^) were found when rice plants were irrigated at 80% of irrigation water requirements (I_80_) and untreated by PGPR.

**Table 10:** Effect of integrative deficit drip irrigation and plant growth promoting rhizobacteria on yield component, grain yield, straw yield and water productivities (G-WP and S-WP) of rice plants grown under saline soil for (SI) 2016/2017 and (SII) 2017/2018 seasons

Source of variation	Grain yield (t ha^−1^)	Straw yield (t ha^−1^)	G-WP (kg m^−3^)	S-WP (kg m^−3^)
Season	NS	NS	NS	NS
(SI) 2017	4.40 ± 0.54a	7.86 ± 1.11a	0.72 ± 0.10a	1.29 ± 0.20a
(SII) 2018	4.37 ± 0.44a	7.79 ± 0.99a	0.71 ± 0.11a	1.26 ± 0.21a
Irrigation	**	**	NS	*
FI	4.85 ± 0.55a	8.32 ± 0.63a	0.79 ± 0.11a	1.35 ± 0.21b
DI	3.93 ± 0.31b	7.33 ± 0.16b	0.80 ± 0.12a	1.49 ± 0.22 a
PGPR	**	**	**	**
^−^PGPR	4.01 ± 0.28b	7.22 ± 0.66b	0.73 ± 0.11b	1.31 ± 0.19b
^+^PGPR	4.77 ± 0.19a	8.43 ± 0.91a	0.86 ± 0.13a	1.52 ± 0.18a
I × PGPR	**	**	**	**
I_100_ × ^−^PGPR	4.46 ± 0.23b	7.86 ± 0.88b	0.73 ± 0.11b	1.28 ± 0.16c
I_80_ × ^−^PGPR	3.56 ± 0.20c	6.58 ± 0.72c	0.72 ± 0.11b	1.34 ± 0.21c
I_100_ × ^+^PGPR	5.24 ± 0.31a	8.78 ± 0.62a	0.85 ± 0.12a	1.43 ± 0.19b
I_80_ × ^+^PGPR	4.30 ± 0.32b	7.98 ± 0.65b	0.87 ± 0.10a	1.62 ± 0.22a

*,**respectively, differences at *p* ≤ 0.05 and *p* ≤ 0.01 probability level, ns indicates no significant difference. Means followed by the same letter in each column are not significantly different according to the LSD test (*p* < 0.05)

## Discussion

Water scarcity is one of the main constraints to agricultural production worldwide, and it is expected to intensify in the future. In arid soil where irrigation is necessary for the production of crops, producers are seeking techniques to save water by increasing the efficiency of irrigation water. Plant growth promoting rhizobacteria (PGPR) is considered one of these strategies and it could play an important role in mitigating the detrimental effects of drought stress on plants. Bacteria strains used in our study [*Bacillu*s *subtilis *subsp. and *Bacillus megatherium*] can produce plant growth promoting substances (PGPs) such as; Indoleacetic acid (IAA) (Loper and Schroth 1986), salicylic acid (Meyer and Abdallah 1978), siderophores (Palli 2005), chitinase (Renwick et al. 1991), cellulose (Andro et al. 1984), phosphate and Zinc solubilization (Rodriguez and Miller 2000; Saravanan et al. 2004) and N2-fixation (Cattelan et al. 1999). Besides, it has antagonistic activity against pathogenic fungi like; *Pythium ultimum*, *Rhizoctonia solani*, and *Fusarium* sp (Koch 1997). The strains also have the capability to live, proliferate sustain life and perform their activities under some adverse environmental conditions such as; temperature, increasing pH, and salt stress. Therefore, *Bacillu*s *subtilis* subsp. and *Bacillus megatherium* are considered as plant growth promoting rhizobacteria (PGPR) and it could use under normal conditions and overcome the negative effects of environmental stresses on some plants (Abdelaziz et al. 2018). The current study has used PGPR as soil application for deficit irrigation DI-stressed rice plants grown under salt stress (ECe = 6.3 dS m^−1^). Inoculating plants with PGPR showed greatly significant positive results for performance growth, water status, stomatal conductance (gs), and chlorophyll fluorescence efficiency, relative chlorophyll content and photosynthetic pigments, antioxidant enzymes and nitrogen contents, yield component, and yields and water productivities of rice plants grown under both DI and saline conditions. In our study, drought stress indirectly inhibited rice growth parameters may be attributed to the drought-induced reduction of cell division and enlargement, resulting in the reduction of shoot length, tillers number plant^−1^, the number of panicles plant^−1^ and shoot dry weight, simultaneously with the reduction of stomatal conductance, water status, chlorophyll fluorescence efficiency, relative chlorophyll content and photosynthetic pigments, as well as antioxidant enzymes and nitrogen contents (Selvakumar and Panneerselvam 2012; Steduto et al. 2012; Abd El-Mageed et al. 2021). On the other hand, inoculation water-stressed rice pants (80% ETc) with PGPR alleviated the deleterious effects of water shortage on rice growth, showing that increased shoot length, tillers number plant^−1^, number of panicles plant^−1^ and shoot dry weight similar to those produced in fully irrigated plants inoculated with PGPR. Also, compared to the untreated plants, inoculation by plant growth promoting bacteria improved rice growth. Rice growth-promoting because of adding PGPR may be linked to the increased micronutrient uptake and affect phytohormones homeostasis. The inoculation effect of our bacterial isolates had a remarkable positive effect on plant growth parameters under stress and non-stress condition. Various studies indicated that PGPRs inoculated plants can take up a higher volume of water and nutrients from rhizosphere soil; the attributes could be useful for the growth of plants under drought stress (Alami et al. 2000). The enhancement of rice growth traits treated with PGPR under water stress may be due to phytohormones like abscisic acid (ABA), indole-3-acetic acid (IAA), salicylic acid, gibberellic acid, cytokinins, and exopolysaccharides which produced by PGPR and help plants to cope with drought stress. A similar trend was reported by Yang et al. (2009), Kim et al. (2012) and Timmusk et al. (2014). The study displayed that rice plants irrigated at 80% ETc and untreated with PGPR produced not only reduction of rice water status (MSI and RWC) but also decreased chlorophyll fluorescence (Fv/Fm and PI) SPAD value, chlorophyll ‘a’ and chlorophyll ‘b’ as well as stomatal conductance, indicating the negative effects of water stress on rice. On the other hand, the canopy-air temperature of rice plants increased by 0.61 °C (Tc–Ta) under water stress (I_80%_) compared to full irrigation. Our results showed that inoculating rice plants with *Bacillu*s *subtilis subsp. and Bacillus megatherium* as a plant growth promoting rhizobacteria (PGPR) stabilized membrane integrity and maintained cell turgor of rice leaves under drought stress. In this concern, increases of tissue RWC and MSI chlorophyll fluorescence (Fv/Fm and PI) SPAD value, chlorophyll ‘a’ and chlorophyll ‘b’ and decreases of canopy temperature (Tc–Ta) as metabolically available water, enabling to maintain tissue health and reflect on the metabolic processes in rice under drought stress. Our results are in line with those reported by Creus et al. (2004), Arzanesh et al. (2011), Liu et al. (2013) and Armada et al. (2014), who reported that PGPR helped plants by increasing leaf water content which was ascribed to the production of plant hormones such as IAA, by the bacteria that improved root growth and formation of lateral roots their by increasing uptake of water, decreased leaf transpiration, improved nutrition and physiology, controlling stomatal closure, and metabolic activities. Also, it was documented that under water stress chlorophyll content (Chl a, and Chl b or SPAD), stomatal conductance, chlorophyll fluorescence (Fv/Fm and PI), photosynthetic parameters as well as water state were increased when plants treated PGPR compared to untreated plants (Wang et al. 2012; Elekhtyar 2015; Samaniego-Gámez et al. 2016; Zhang et al. 2019). In the present work the reduction of antioxidant defense system (e.g., peroxidase (PO), polyphenol oxidase (PPO), total phenol), N% (leaves), and N% (grains) under drought stress may be due to the influences of drought stress on the availability and transport of nutrients, as soil nutrients are carried to the roots by water. Our results are in line with those of Selvakumar and Panneerselvam (2012), Abd El-Mageed et al. (2017) and Semida et al. (2021a). They reported that water stress reduces nutrient diffusion and mass flow of water-soluble elements such as nitrate, K, Ca, Mg, and Si. Moreover, drought induces free radicals affecting antioxidant defenses such as superoxide radicals, hydrogen peroxide, and hydroxyl radicals. However, our study exhibited that the negative effects on antioxidant defense system (e.i., peroxidase (PO), polyphenol oxidase (PPO), and total phenol), N% (leaves), and N% (grains) of water-stressed rice were alleviated by inoculated by PGPR, thereby enhanced antioxidant enzymes and N% contents (leaves and grains). In these concerns, Yogendra et al. (2015) reported that PGPR mitigates oxidative damage in rice plants grown under drought by increasing plant growth and activating antioxidant defense systems, thereby enhancing the stability of membranes in plant cells. Additionally, PGPR increased rice biomass production grown under drought stress. Enhancement of the plant dry biomass is a positive criterion for drought tolerance correlates with an increase of rice yields (Yogendra et al. 2015). Our strains have the ability to fix N, thus led to an increase in N uptake in leaves and grains These positive results in response to PGPR application may be related to PGPR and regulated the redistribution and uptake of N, besides restoration of photosynthetic efficiency (Rodriguez et al. 2004; Anjum et al. 2007), and more metabolites required for rice growth. Drought stress (I_80%_) positively decreased rice yield attributes (e.g., panicle length, panicle length, panicle weight, grains number panicle^−1^, and 1000 grain weight) and yields (grain and straw) compared to fully irrigated plants (I_100%_). The reduction in yield components under water stress may be due to the decreases in growth, stomatal conductance, chlorophyll content, water status, N uptake, and photosynthesis efficiency of plants (Quampah et al. 2011; Pejic et al. 2011). Consequentially, the reduction in panicle length, panicle length, panicle weight, grains number panicle^−1^, and 1000 grain weight decreased the yield of grain and straw. In these concern, Pantuwan et al. (2002), Wu et al. (2011), Kumar et al. (2014) and Yang et al. (2019) reported that water stress could cause spikelet degenerate, spikelet sterility, and grains number reduce unfilled grain No. increase, and 1000-grain weight and yield reduce. The G-WP values were not affected significantly by the irrigation quantity where S-WP values were significantly affected and the highest values for G-WP and S-WP were recorded under I_80%_ treatment. A similar trend was reported by Semida et al. (2014) and Rady et al. (2021a, b). In general, according to the results of various experiments, lower water application provides higher WP values (Rady et al. 2021a; Semida et al. 2021b). Li et al. (2001) indicated that limited irrigation in wheat during the growing season could significantly increase WP. Abd El-Mageed et al. (2018) and Agami et al. (2018) found that the highest values of WUE for sorghum and wheat were recorded via low moisture conditions (60% of Class A pan evaporation). Results of the current study indicate that inoculation rice plants by PGPR enhanced yield, yield components, and G-WP and S-WP irrespective of irrigation treatment, and the higher rice values were noted when rice plants irrigated well and inoculated by bacillus subtilis, and bacillus megatherium strains. This could be as a result of enhancing the survival, and growth yield, yield components, and G-WP and S-WP under PGPR inoculation by improving morpho-physiological responses, chlorophyll efficiency, plant water status, providing higher protection for plant tissues and thus led to an increase in yields and water productivities. This result is found to be in harmony with Hussain et al. (2014) for wheat, Kang et al. (2014) for soybean, Cohen et al. (2009) for maize, Cassán et al. (2009) and García de Salamone et al. (2012) for rice. They concluded that the application of PGPRs in plants increased yield and alleviated water stress by various mechanisms such as; reduced oxidative damage, increased proline, abscisic acid, auxin, gibberellin, and cytokinin content; improved vegetative growth, water status of the plant, photosynthetic capacity and nutrients status; enhanced physiological and biochemical attributes.

## Conclusion

Exposure of rice plants to drought stress positively reduced, physiological responses, RWC%, MSI%, antioxidant enzymes (e.i., peroxidase (PO), polyphenol oxidase (PPO), total phenol), N% (in leaves and grains), growth attributes, grain, and straw yields and increased canopy temperature the of rice plants. However, inoculation rice plants with PGPR could mitigate the deleterious effects of water stress by enhancing leaf photosynthetic pigments, chlorophyll fluorescence, SPAD value, stomatal conductance, plant water status, antioxidant enzymes, plant growth, yields, and WP and reduce plant canopy temperature. Depending on the obtained results it could be summarized that the treatment (I_100_ × ^+^PGPR) is the most suitable for obtaining the highest grain and straw yields. Under water deficit, the application of (I_80_ × ^+^PGPR) treatment was found to be a favorable strategy to save 20% of the applied irrigation water, providing the same rice yield. Our results suggest that PGPR applications may find value as anti-abiotic stresses for improving rice growth and productivity under drought stress.

## Data Availability

The datasets used and/or analyzed during the current study are available from the corresponding author on reasonable request.
